# Can action observation modulate balance performance in healthy subjects?

**DOI:** 10.1186/s40945-018-0053-0

**Published:** 2019-01-22

**Authors:** Roberto Gatti, Elisabetta Sarasso, Mattia Pelachin, Federica Agosta, Massimo Filippi, Andrea Tettamanti

**Affiliations:** 1 Laboratory of Movement Analysis, San Raffaele Scientific Institute, via Olgettina 58, 20132 Milan, Italy; 2 Degree Course in Physiotherapy, Vita-Salute San Raffaele University, Milan, Italy; 3 Neuroimaging Research Unit, Institute of Experimental Neurology, Division of Neuroscience, San Raffaele Scientific Institute, Vita-Salute San Raffaele University, Via Olgettina, 60, 20132 Milan, Italy; 4 Rehabilitation Department, San Raffaele Hospital, Milan, Italy; 5 Department of Neurology, Institute of Experimental Neurology, Division of Neuroscience, San Raffaele Scientific Institute, Vita-Salute San Raffaele University, Milan, Italy; 6 Physiotherapy Unit, Humanitas University and Humanitas Clinical and Research Center, Rozzano, Italy

**Keywords:** Action observation, Action observation training, Balance, Postural control

## Abstract

**Background:**

Action observation activates brain motor networks and, if followed by action imitation, it facilitates motor learning and functional recovery in patients with both neurological and musculoskeletal disorders. To date, few studies suggested that action observation plus imitation can improve balance skills; however, it is still unclear whether the simple repetitive observation of challenging balance tasks is enough to modify postural control. Thus, the primary aim of this study was to investigate whether repetitive action observation of balance exercises without imitation has the potential to improve balance performance; the secondary aim was to estimate the different training effects of action observation, action observation plus imitation and balance training relative to a control condition in healthy subjects.

**Methods:**

Seventy-nine healthy young adults were randomly assigned to 4 groups: action observation, action observation plus imitation, balance training and control. The first three groups were trained for about 30 minutes every day for three weeks, whereas the control group received no training. Center of pressure path length and sway area were evaluated on a force platform at baseline and after training using posturographic tests with eyes open and closed.

**Results:**

As expected, both action observation plus imitation and balance training groups compared to the control group showed balance improvements, with a medium to large effect size performing balance tasks with eyes open. Action observation without imitation group showed a balance improvement with eyes open, but without a significant difference relative to the control group.

**Conclusions:**

Both action observation plus imitation and balance training have similar effects in improving postural control in healthy young subjects. Future studies on patients with postural instability are necessary to clarify whether AOT can induce longer lasting effects. Action observation alone showed a trend toward improving postural control in healthy subjects, suggesting the possibility to study its effects in temporarily immobilized diseased subjects.

## Background

Since childhood, humans demonstrate an extraordinary ability to predict another individual’s movement, to adapt their motor plans according to another person’s intention and to learn from other people’s behaviour [1]. The neural substrate underlying observation, understanding and imitation of movements is the mirror neuron system [2]. The visual information is primarly processed in the visual system and then projected to the mirror neuron system, which is involved in understading the meaning of actions [3, 4].The mirror neuron system is mainly located in the fronto-parietal regions and plays an important role in building a motor memory modulating the motor behavior of the observer [3, 4].

Many studies have demonstrated that the observation of meaningful actions also stimulates the activity of motor and motor-related networks without any movement execution. Action observation (AO) can be interpreted as a form of “motor simulation” [5] evoking an internal representation of the observed movement also called “motor resonance” [6], which involves motor brain areas similar to those usually firing during movement. As suggested by Jeannerod, mental representation of movement contains many charachteristics of the future action such as the goal, the motor program and its consequences [5, 7]. AO does not only share a common brain pattern with motor execution but it can also facilitate movement performance. Most pieces of evidence have showed that the observation of goal-directed actions stimulates a mental re-enactement of the observed actions and facilitates action imitation [8].

In the last few years, AO followed by imitation of the observed exercises (AOT) has emerged as an alternative and complementary rehabilitative approach in patients with central nervous system lesions, even in the presence of poor residual movements, taking advantage of the possibility to exercise motor networks offline [8]. Several studies have demonstrated the effect of AOT in promoting motor learning and functional recovery after stroke [9], in people affected by Parkinson’s disease [10, 11], in children with cerebral palsy [12], and also in post-surgical orthopedic patients [13].

Increasing attention has been addressed to the role of AOT in improving postural control [1]. In particular, three studies showed an improvement on balance performance in chronic stroke subjects after AOT of walking abilities [14–16]. A possible neurophysiological explanation could be that locomotor adaptation is modulated by observing actions of others such as walking in challenging environment: the greater the postural sway observed, the greater the postural countermeasures adopted to adapt locomotor behaviour [17]. Moreover, Bhatt and Pai suggested that the mere observation of a slippery walking can induce awareness during real walking, thus promoting a greater stability [18]. Taube et al. also investigated the effect of AO associated with motor imagery on balance performances [19]. In this latter study, the authors showed that both motor imagery and AO associated with motor imagery of postural exercises can reduce postural sway during stance with and without unexpected externally perturbations [19].The same authors showed that brain regions known to be involved in the execution of balance tasks are also active during imagination and AO plus imagination of balance tasks [20]. This suggests the idea that even AO alone can be successfully used to improve postural control and to reduce the risk of falls in temporarily immobilized patients.

In order to understand the real contribution of AO alone on postural stability, the main aim of the present study was to assess whether a multi-session training consisting of AO of balance tasks can improve postural sway. Moreover, we compared the effect of AO with other well-known training approaches such as AOT and balance training (BT). We expected subjects trained with AOT and BT to improve balance performance by reducing postural sway; we also hypothesized that AO could play a role in modifying postural behaviour, even if with a smaller effect size relative to AOT and BT.

## Methods

### Participants

Seventy-nine healthy young subjects (39 females, 40 males, mean age 21.39 years±SD 1.73), recruited among University students, participated in this study (Table 1). Inclusion criteria were the following: 1) age < 30 years; 2) normal or corrected to normal vision; 3) absence of neurological and orthopedic disorders; 4) absence of specific balance skills due to competitive sports. The current work has been carried out in accordance with The Code of Ethics of the World Medical Association (Declaration of Helsinki) for experiments involving humans; informed consent was obtained from all subjects.

**Table 1:** Demographic characteristics of the participants and posturographic variables at baseline

	CO	AO	BT	AOT	p
Sample size (n)	20	20	19	20	–
Age (years)	22.00	21.00^a^	21.00	21.50	0.03
[range]	[21–28]	[20–24]	[19–26]	[19–23]	
Weight (kg)	66.50	60.00	67.00	70.00	0.59
[range]	[47–83]	[50–86]	[50–85]	[50–87]	
Height (cm)	175.00	171.00	173.00	174.00	0.68
[range]	[158–190]	[159–192]	[163–193]	[160–195]	
BMI (kg/cm^2^)	21.28	20.86	21.14	21.98	0.38
[range]	[18.82–24.78]	[19.05–26.54]	[18.81–24.72]	[17.30–26.26]	
Balance measures					
CoP Sway Path Length eyes open (mm)	767.02 ± 120.37	818.19 ± 99.64	802.61 ± 77.72	766.08 ± 86.32	0.28
CoP Sway Path Length eyes closed (mm)	1139.35 ± 226.96	1138.92 ± 593.31	1213.40 ± 219.62	1122.54 ± 252.01	0.63
CoP Sway Area eyes open (mm^2^)	1945.20 ± 675.74	2180.27 ± 593.37	1947.27 ± 472.17	1885.15 ± 427.41	0.36
CoP Sway Area eyes closed (mm^2^)	4343.04 ± 1742.13	4168.93 ± 1404.93	4783.75 ± 1997.22	4156.97 ± 1559.61	0.79

Values are mean [range] or ± standard deviation. *P* values referred to Kruskal Wallis test. ^a^ = AO group is younger than CO group according to post-hoc comparisons

*AO* action observation group, *AOT* action observation training (AO plus imitation) group, *BT* balance training group, *BMI* body mass index, *CO* control group, *CoP* center of pressure

### Training design

The participants were randomly assigned to 4 groups: AO (action observation without imitation), BT (balance training), AOT (action observation plus imitation), and CO (control group). A computerized random list generator (random.org) provided the randomization list. All groups were trained for three weeks, five days per week (Monday to Friday), except for the CO group which received no treatment.

**Fig. 1:**
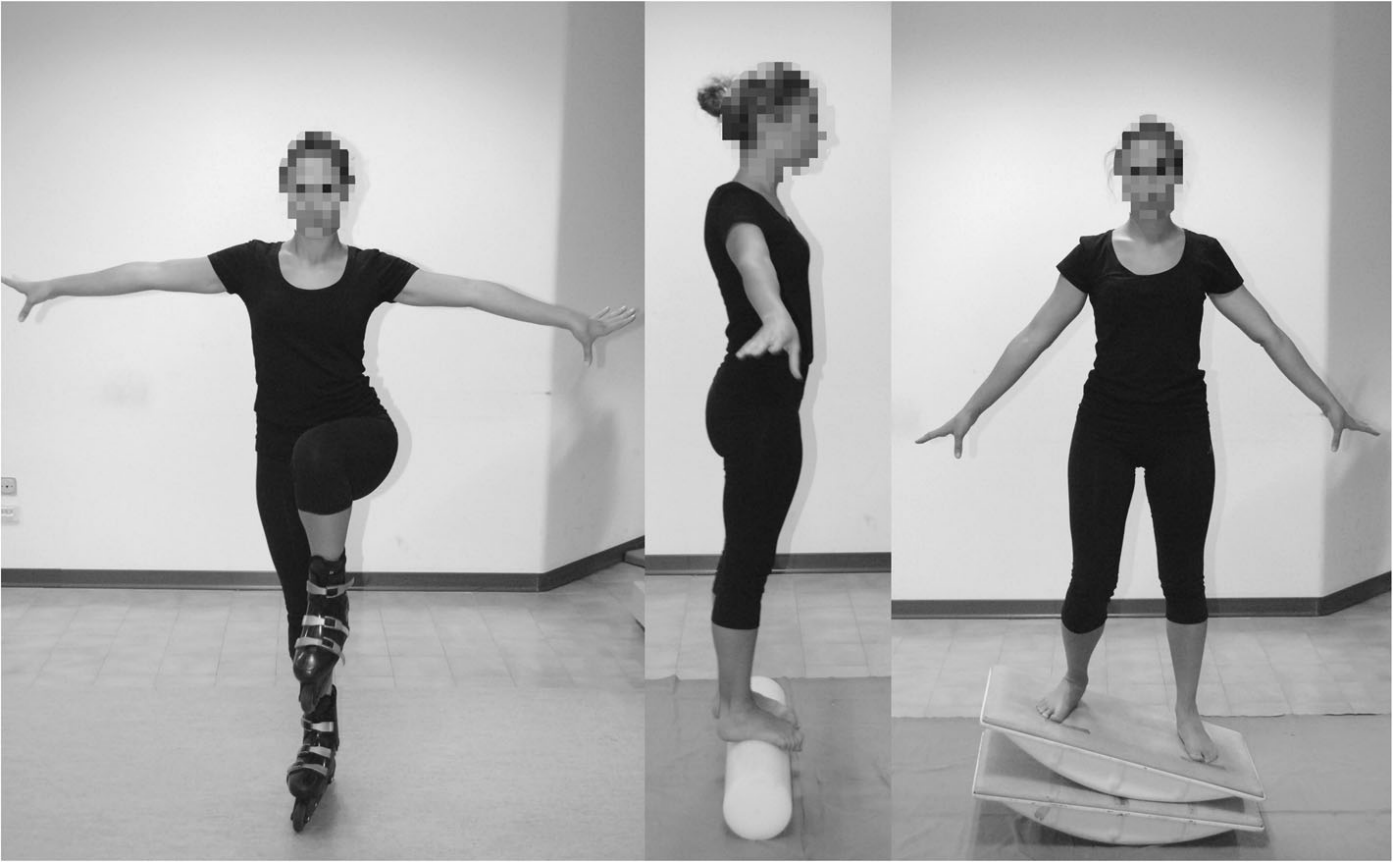
The figure shows three examples of balance exercises performed by balance training (BT) and action observation training (AOT) groups

A total of 30 videos of challenging balance exercises performed by professional athletes, for example walking on a balance beam with eyes closed, standing on one leg on a trampoline, standing on a wobble board (see Fig. 1 for other examples), were used for AO and AOT groups. Each video provided a single exercise in both sagittal and frontal perspectives and lasted 5 min. For each video, two versions (with a male and a female as actors) were available and were administered in accordance with the gender of participants. AO sessions consisted of carefully watching 4 different videos, with resting periods of 4 min between them. AOT participants watched the same 4 videos as AO group and, after each video, they imitated the exercise for 3 min. BT participants watched 4 different videos, showing landscapes with no motor content; after each video, they were asked to perform the same balance exercise seen by AO/AOT groups following the verbal instructions of a physiotherapist. Each exercise was performed for 3 min. Considering videos, exercises and rest, all the groups underwent sessions lasting 32 min.

The same training was performed for two consecutive days, with increasing level of exercise difficulty. Each subject received individual training sessions in a quiet room. The same physiotherapist, blinded to baseline assessement, trained subjects during the exercises.

### Balance performance assessment

The subjects’ displacement of the center of pressure (CoP) was evaluated at baseline (T0, the day before the first training session) and at the end of the training period (T1, the day after the last training session) using posturographic tests executed on a force platform (KISTLER mod. 9286A, CH). Test acquisitions were performed using a sampling frequency of 100 Hz. Participants were instructed to stand on the force platform and to look forward in upright position with arms at their sides. The balance tests consisted in the maintenance of 4 static positions (both eyes open and eyes closed): 1) bipedal stance; 2) single right stance; 3) single left stance; 4) bipedal stance on a foam pad. Bipedal stances lasted 30 s, whereas single leg stances lasted 15 s. Moving arms to recover balance was not allowed. At the end of each test, the subjects were allowed to sit on a chair for about 3 min to have a rest, and each test was repeated twice. The values of CoP oscillations (CoP Sway Path Length and Area) were analyzed using the software “Sway 1.4.1”, BTS S.p.A; the best performance, defined by the lower value of CoP Sway Path Length or Area was considered.

### Statistical analysis

CoP Sway Path Length and Area were expressed in mm and mm^2^ respectively. Considering the relatively small sample size and the non-normal data distribution (assessed using Kolmogorov Smirnov and Shapiro Wilk tests), non parametric statistic was used. T0 variables were compared between groups using the Kruskal Wallis test followed by Mann Whitney post-hoc comparisons. The Wilcoxon Signed Ranks test was used to assess changes overtime in each group. Differences between T0 and T1 were compared between groups using Kruskal Wallis and Mann Whitney tests. Correction for multiple comparisons was performed using Benjamini–Hochberg procedure to adjust for False Discovery Rate (alpha level = 0.05, type I error). Statistical significance was accepted for values of *p* <  0.05. The effect size (standardized mean difference) of each treatment was then calculated using Hedges’ *g* approach. Effect size was considered small if the score was 0.2, medium if the score was around 0.5 and large with a score around or above 0.8 [21]; the effect was statistically significant if the confidence interval did not cross the zero. All data were analysed using the software SPSS 23.

## Results

The groups were similar at baseline in terms of demographic variables (height, weight and BMI), except for the age that was relatively higher in CO group relative to AO group. Moreover, no significant differences in balance performance were found between the 4 groups at T0 (see Table 1).

### Within-group changes

All the 3 trained groups showed improvements in balance performance at T1 relative to T0, while the CO group did not (Table 2). AO group showed a reduced CoP Sway Area during eyes open balance tasks. BT group showed a reduced CoP Sway Area during eyes open balance tasks and also a reduced CoP Sway Path Length during balance tasks both with eyes open and eyes closed. AOT group improved both CoP Sway Path Length and Area during eyes open balance tasks.

**Table 2:** Table shows mean and standard deviation values of Center of Pressure (CoP) Sway Path Length and Sway Area before (T0) and after (T1) training in each group for posturographic tests with eyes open and closed

	CO			AO			BT			AOT				p^#^ CO vs AO	p^#^ CO vs BT	p^#^ CO vs AOT	p^#^ AO vs BT	p^#^ AO vs AOT	p^#^ BT vs AOT
Balance Test	T0	T1	p^§^	T0	T1	p^§^	T0	T1	p^§^	T0	T1	p^§^	p*						
CoP Sway Path Length eyes open (mm)	767.02 ± 120.37	783.96 ± 119.73	0.65	818.19 ± 99.64	784.62 ± 116.72	0.10	802.61 ± 77.72	750.14 ± 71.03	0.01	766.08 ± 86.32	710.61 ± 65.56	< 0.001	0.15	0.33	0.04	0.04	0.44	0.44	0.95
CoP Sway Path Length eyes closed (mm)	1139.35 ± 226.96	1139.79 ± 178.20	0.97	1138.92 ± 593.31	1091.31 ± 220.96	0.22	1213.40 ± 219.62	1088.13 ± 165.50	0.004	1122.54 ± 252.01	1057.50 ± 179.55	0.12	0.36	0.57	0.13	0.53	0.53	0.66	0.57
CoP Sway Area eyes open (mm^2^)	1945.20 ± 675.74	1924.34 ± 559.98	0.82	2180.27 ± 593.37	1936.18 ± 481.36	0.02	1947.27 ± 472.17	1845.19 ± 508.95	0.08	1885.15 ± 427.41	1682.13 ± 468.50	0.01	0.41	0.38	0.62	0.38	0.64	0.76	0.64
CoP Sway Area eyes closed (mm^2^)	4343.04 ± 1742.13	4237.84 ± 1549.17	0.91	4168.93 ± 1404.93	3907.92 ± 1730.11	0.19	4783.75 ± 1997.22	4045.85 ± 1474.02	0.02	4156.97 ± 1559.61	3690.60 ± 1494.24	0.22	0.50	0.75	0.672	0.75	0.67	0.78	0.75

p^§^ referred to Wilcoxon test; p* referred to Kruskal Wallis test; p^#^ referred to Mann Whitney test

p values were adjusted for multiple comparisons using the linear step-up procedure of Benjamini and Hochberg (False Discovery Rate controlling adjustment)

*AO* action observation group, *AOT* action observation training (AO plus imitation) group, *BT* balance training group, *CO* control group, *CoP* Center of Pressure

### Between-group changes

The comparison of balance task changes between groups over time showed significant differences in favour of the AOT and BT groups compared to the CO group (Table 2). Specifically, the BT group showed an improved CoP Sway Path Length performing balance tasks with both eyes open and closed, while AOT group only during open eyes balance tests. Relative to the CO group, AO did not show any significant change overtime.

### Effect size analysis

**Fig. 2:**
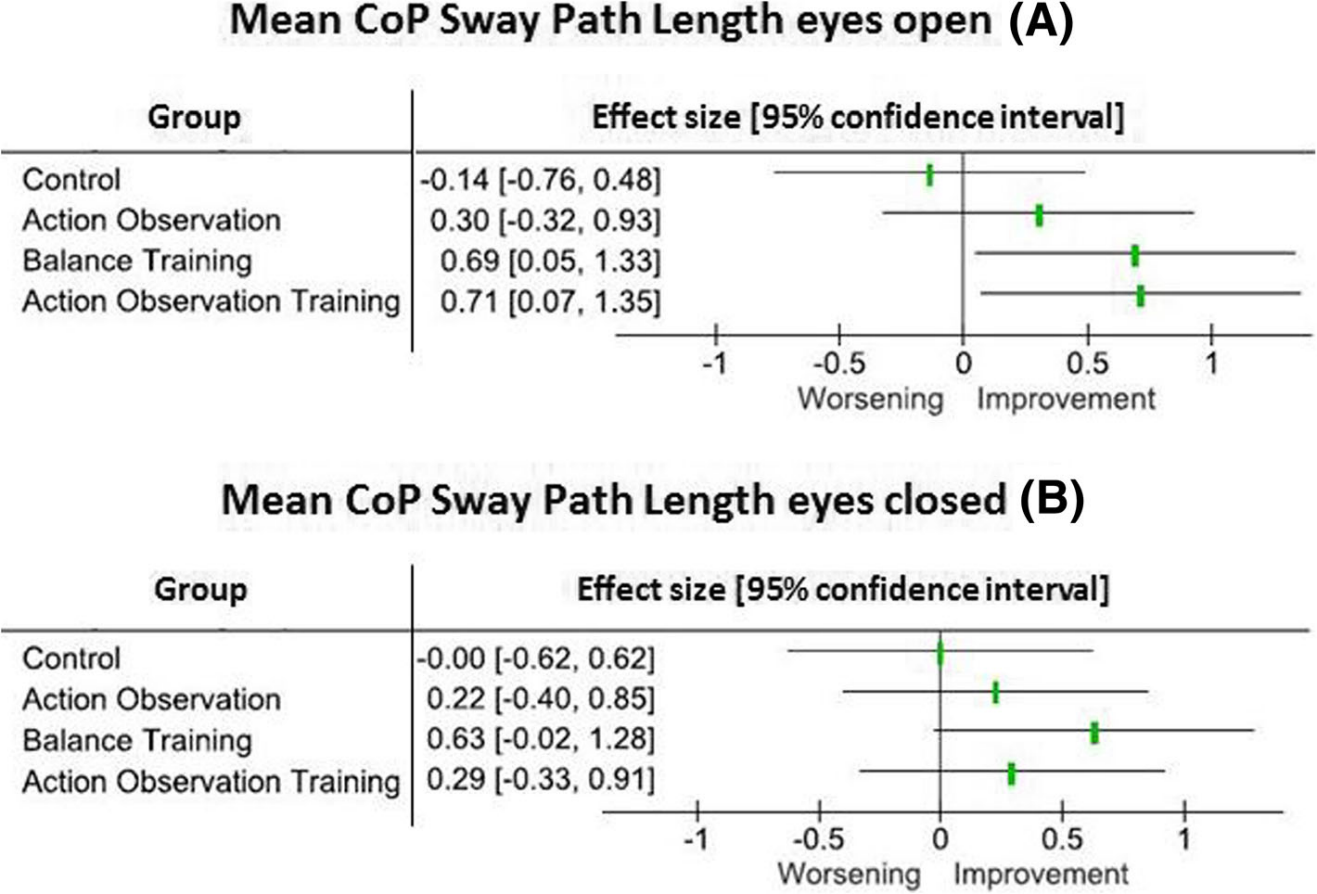
The figure shows the effect size of each training on Center of Pressure (CoP) Sway Path Length during eyes open (**a**) and eyes closed (**b**) balance tests

As illustrated in Fig. 2, we found that AOT and BT showed a significant and medium to large effect size (0.7 [0.05–1.35 C.I.]) by reducing CoP Sway Path Length during eyes open tasks; the AO group showed a small to medium effect size, which however was not statistically significant (0.3 [− 0.32–0.93 C.I.]. Performing eyes closed balance tasks, no group achieved a significant effect, with a trend toward a significant and medium/large effect size in the BT group (0.6 [− 0.02–1.28 C.I.]. No effect was found in the control group in both balance tasks.

## Discussion

This study showed that AOT and BT can modify postural control by reducing postural sway in healthy young subjects. As expected, both AOT and BT showed also a significant effect size on balance performance, with a medium to large effect performing tasks with eyes open [16, 22]. AO without imitation showed significant reduction of CoP Sway Area during eyes open balance tasks, but without reaching a significant difference relative to the control group or a significant effect size. Certainly, AOT is expected to be more effective in improving motor performance relative to AO alone; however, the small effect induced by AO might become clinically important if obtained in patients with postural instability. Thus, our results suggest the possibility to study AO effects to train motor learning of balance tasks in temporarily immobilized diseased subjects. The mere observation of actions is actually part of the complex mechanism of motor learning: AO can evoke an internal representation of the observed movement [7, 23], strengthening the formation of a motor memory and facilitating the subsequent motor performance [1, 10]. Indeed, AO activates brain areas similar to those recruited performing the given action, such as the motor brain network and the mirror neuron system [6]. These areas are usually engaged during the acquisition of new motor skills and particularly during the motor learning of goal-directed behaviours [10].

Previous studies suggested that the observer is able to generate predictions about motor behaviours of others by covertly simulating the observed action [24]. Moreover, humans can easily detect movement errors and also adapt their behaviour in order to avoid the observed errors: for instance, the observation of a person that is about to fall induces a state of awareness which can reduce risk of fall when the observer walks on unstable surfaces [18]. Most recently, Tia et al. confirmed the presence of a “contagion effect” which consists of a contagious postural reaction following the observation of human imbalance; interestingly authors demonstrated that the postural response can be modulated by habituation, suggesting a possible learning effect [25, 26]. The size of the observed postural sway is also correlated with the subsequent motor response of the observer during the same balance task [17]. For this reason, a crucial issue is the difficulty level of the proposed training: not only the executed exercises must be challenging in order to obtain balance improvement, but also the observed perturbations need to be large to obtain an aftereffect in the observer [17]. Thus, we proposed videos showing complex balance exercises, in order to stimulate the attention about details of different postural reactions and balance strategies used to overcome destabilizations. Interestingly we tested subjects in different balance conditions relative to those observed, anyway obtaining an improvement of postural sway. These results suggest that the mere observation of another person in a challenging environment is enough to increase postural awareness and stimulate postural countermeasures also performing different balance tasks.

Interestingly, it would seem that subjects involved in AO and AOT performed better in tests with eyes open. As known, the vision is an important mechanism that works together with proprioceptive and vestibular feedbacks to maintain balance [27]; in particular, visual information is continuously integrated by the cerebellum in order to correct movement errors and detect the better timing of execution. Moreover, the visual stimulus is known to be salient in enhancing attentive functions performing actions [28], thus AO can be interpreted as an attentional strategy that could improve externally focused attention during the execution of attention-demanding tasks such as balance exercises. This point is particularly interesting, considering recent evidence showing that the risk of falls in elderly subjects is strongly correlated with the performance at cognitive tests. In particular, executive, attentive and visuospatial alterations contributed to augment risk of falls in elderly subjects together with motor difficulties such as increased body sways, low reaction time and gait speed, weak muscle strength, and poor visual contrast [29]. Thus, it would be interesting to study the possible effects of AO and AOT in stimulating cognitive networks in elderly people to improve balance and reduce risk of falls.

Moreover, recent studies suggested that also motor imagery and AO associated with motor imagery can improve balance performance by reducing postural sway during stance with and without perturbations [19]. These two strategies can also induce functional brain activation in regions known to be involved in the execution of balance tasks [20]. More recent evidence suggested that AO and motor imagery can be considered as different parts of the same paradigm [30] and their effects may influence each other in a very specific way [31]: motor imagery can modulate the effects of AO on motor learning [32] and greater brain activations in cortical-subcortical networks were found in the AO plus motor imagery condition relative to AO and motor imagery separately [20, 33, 34]. Considering these findings, future studies are needed to confirm the potential superiority of a combined AOT-motor imagery approach to improve postural instability.

This study is not without limitations. Firstly, we proposed balance exercises and tests involving mainly feedforward mechanisms. Further studies are needed to analyse also feedback balance strategies. Moreover, this is a pilot study, the number of subjects is relatively small and no information about the long-term effects of trainings on postural sway is available. A possible advantage of AOT is that it could potentiate motor learning providing a longer lasting effect relative to motor training alone [8]. Thus, future studies should investigate the long-term effects of this approach on balance performances. We did not find any significant difference between different trainings, but we included only healthy young subjects whithout any balance impairment. Future studies should involve a population of patients with postural instability to investigate if results reach a clinical impact. AO without imitation might be proposed in temporarely immobilized diseased patients to promote learning of motor strategies useful to perform future motor esercises. However further studies are needed to probe this hypothesis. Finally, it would be interesting to use functional magnetic resonance imaging in order to understand the neural mechanisms underlying our findings.

## Conclusion

According to our findings both AOT and BT have similar effects in improving postural control in healthy young subjects, with a medium to large effect size performing balance tasks with eyes open. AO alone showed the possibility to modulate motor behaviours during balance tasks in some healthy young subjects, but without significant changes relative to the control group. Our findings also showed no significant differences between AO, AOT and BT effects on balance performance in healthy young subjects. However, further studies are needed to: i) investigate the effects of AO to promote learning of motor strategies useful to perform future motor esercises in temporarily immobilized diseased subjects; ii) define if AOT of balance exercise relative to BT has greater effect to improve postural control earlier and with more long-lasting results in patients with postural instability.

## Abbreviations

AO: Action observation (without imitation)
AOT: Action observation training (AO plus imitation)
BT: Balance training
CO: Control group
CoP: Center of pressure
T0: Baseline
T1: After three weeks of training
